# Accuracy of Implant Casts Generated with Conventional and Digital Impressions—An In Vitro Study

**DOI:** 10.3390/ijerph15081599

**Published:** 2018-07-27

**Authors:** Paulo Ribeiro, Mariano Herrero-Climent, Carmen Díaz-Castro, José Vicente Ríos-Santos, Roberto Padrós, Javier Gil Mur, Carlos Falcão

**Affiliations:** 1 Porto Dental Institute, 4150-518 Oporto, Portugal; ribeiropaulo1@gmail.com (P.R.); mariano@herrerocliment.com (M.H.-C.); 2 Department of Periodontics and Dental Implants, University of Seville, 41009 Seville, Spain; carmmaria@hotmail.com; 3 Barcelona Dental Institute, 08034 Barcelona, Spain; robertopadros@hotmail.com; 4 Technological Health Research Center, Biomaterials of the Faculties of Medicine and Dentistry, International University of Cataluña, 08034 Barcelona, Spain; xavier.gil@uic.es; 5 Porto Dental Institute, Faculty of Health Sciences, Fernando Pessoa University, 4150-518 Oporto, Portugal; cfalcao@ufp.edu.pt

**Keywords:** digital dental impression, accuracy of implant casts

## Abstract

Purpose: The aim of this study was to compare the accuracy of digital dental impressions with the accuracy of impressions obtained via conventional techniques. Methods: Two different master models were created, one with parallel implants (model 1) and the other with non-parallel implants (model 2). These reference master models included 4 Klockner KL RP implants (Klockner Implant System SA, Barcelona, Spain), which were juxta-placed and equidistant in the intermentoneal region. In model 1 the implants were placed parallel to each other, whereas in model 2 the implants were placed such that there was a divergence angle of 15° between the more distal implants, and a convergence angle of 15° between the two central implants. A total of four types of impressions were obtained from model 1 (four groups, *n* = 10 each), including closed tray impressions with replacement abutments; open tray impression groups for dragging copings, without splinting; open tray impressions for ferrules; and impressions obtained using the 3M^TM^ True Definition Scanner system. For model 2 three groups were created (three groups, *n* = 10 each), including closed tray impressions with replacement abutments; open tray impression for dragging copings, without splinting; and impressions obtained using the 3M^TM^ True Definition Scanner system. The master models and the models obtained using conventional methods were digitalized in order to compare them via an extraoral high-resolution scanner (Imetric IScan D104i, Porretruy, Switzerland). The STL (Stereo Lithography (format for transferring 3 dimensional shape information)) digital values were loaded into reverse-engineering software and superimposed with their respective STL master models in order to evaluate deviations in three dimensions. We then analyzed the squares of the deviations in the three axes and evaluated the median and the sum of the deviation square. Statistical analysis was performed using the IBM Corp. Released 2016. IBM SPSS Statistics for Windows, Version 24.0. Armonk, NY: IBM Corp. The normality of the distributions was analyzed according to a Kolmogorov-Smirnov test. The median comparison was performed using the differences between the medians, analyzed using non-parametric Kruskal-Wallis and Mann-Whitney tests with a significance level of *p* < 0.05. Results: For model 1, the deviations of the digital impressions were smaller than those associated with the conventional techniques. The sum value in group D was 1,068,292, which was significantly lower than those of groups A, B, and C, which were shown to be 2,114,342, 2,165,491, and 1,265,918, respectively. This improvement was not observed when using model 2, however, where the conventional techniques yielded similar results. Group F simultaneously presented the lowest total square sum of the three deviations (1,257,835), indicating a significantly higher accuracy for this group in model 2, while the sum values were 1,660,975 and 1,489,328 for groups E and G, respectively. Conclusion: Digital impressions of full-arch models were able to achieve the accuracy of conventional impressions in an in vitro model. Nevertheless, further in vivo studies are needed to validate these in vitro results.

## 1. Introduction

Dental implants are an alternative treatment to conventional dentures for patients who have lost their teeth, and they can provide long-term stability and comfort [1,2]. They require an initial operation to facilitate the fixation of the dental implant into the bone, then in a second step a prosthesis is connected to the screw. It is generally accepted that a suitable surgical technique and a passive fit of the prosthesis are key to the long-term success of the treatment [3,4,5,6,7,8]. From a biomechanical perspective, a poor-fitting prosthesis connected to the dental implant can increase stresses and tensions in the bone surrounding the dental implant, leading to marginal bone loss, which may in turn lead to implant loosening and subsequent implant failure [4,5,6,7,8,9,10].

A key element that dictates the passive adjustment of the prosthesis is the precision of the working model, which in turn depends directly on the precision of the impression technique [11]. Some of the possible causes of a non-passive adjustment of the prosthesis are excessive inclination and the depth of the implants [11], as well as the potential inaccuracies of current impression materials and techniques. In general, these include dimensional changes of the impression materials, mainly the expansion of the working model material, contraction of the cast metal, dimensional changes in the wax and acrylic registers, and dimensional changes in the coating materials [11,12]. It is crucial to replicate the implant to be placed in the patient with the highest precision with regard to the working model and the impression, so as to ensure the capacity for passive adjustment of the connection [13].

Several impression techniques have been used for the construction of master models designed to enable the correct clinical adjustment of the structure. A recent systematic review on the influence of the accuracy of impressions on implants showed that splinted copings are superior to non-splinted copings in both partially and totally edentulous patients. The need to splint the coping posts has been advocated in some studies, while others have not reported relevant aspects in the splinting process [3,7,12,14]. It has also been suggested that open tray techniques are better than closed tray techniques in totally edentulous patients, although no significant differences were found in partially edentulous patients [15].

Digital dentistry is currently revolutionizing the way laboratory scientists and clinicians communicate, because digital impression scanners eliminate the procedures associated with tray selection, dispensing and placing the impression material in the mouth, disinfecting it, and sending the physical impression to the laboratory. Furthermore, digital impressions offer enhanced patient comfort and greater efficiency, because such impressions can be digitally controlled and electronically stored. 

The conventional physical impression with trays and materials (alginates, silicones, polyethers) represents a moment of discomfort for the patient [16,17]; this is particularly the case with sensitive subjects, for example those with a strong gag reflex [16]. In addition, it can be difficult for the clinician, especially in the case of technically complex impressions (for example the fabrication of a long-span implant-supported reconstruction) [12,14]. The optical impression with Intraoral Scanners (IOS) solves all these problems: it is well-tolerated by the patient, since it does not require the use of conventional materials, and is technically easier for the clinician [16,17,18,19,20,21,22]. The use of an intra oral scanner allows an immediate determination of the impression quality; virtual three-dimensional (3D) models of patients are obtained, which can be saved on a computer without physically pouring a plaster model [19,20,21,22,23]. This saves time and space, and provides the ability to easily send the models to the laboratory using e-mail, reducing time and costs [16,17]. The clinician can save money each year on the purchase of impression materials, the fabrication of individual trays, and on the casting and shipping of plaster models, since it is possible to store virtual models of patients without having to allocate a space within the clinic [16,17,18,19,20,21,22,23]. Not least, the clinician can have a powerful marketing tool for more effective communication with the patient [16,17].

Due to the novelty of digital impression systems, research pertaining to their application in the field of dental implants is limited to a series of cases. To date, discrepancies between different implant impression techniques have been generally measured from a mechanical perspective [24]. As a result of the introduction of computer-aided design/computer-aided manufacturing (CAD/CAM) technology in dentistry, the preliminary digitalized models can be compared and overlapped, facilitating the relative assessment of the digital recording modalities and their associated deviations [13,25]. The objective of the current study was to compare the accuracy of digital impressions and different conventional printing techniques in the context of dental implants.

## 2. Materials and Methods

Using the program N Query Advisor 4.0 with a level of significance of 0.05 and four study groups, based on the article of Vandeweghe S et al. [26] and a power of 80%, we obtained a value of six participants per group (*n* = 6). Given that there was the possibility that the standard deviation of our work was a little wider, we decided to raise the number the number of participants to *n* = 10; As it is an in vitro study, there were no patients exposed to unnecessary risks, and it could be safely increased from 6 to 10, in order to improve the reliability and precision of the results. The same number per group was used in the study of Papaspyridakos P. et al. [11].

### 2.1. Model Preparation

Initially, in order to prepare the reference models two master models were constructed: one with parallel implants (model 1) and another with angled implants (model 2). These reference master models were made of a polyurethane resin (POLIUROCK, Metalor Technologies SA, Neuchatel, Switzerland) and included 4 Klockner KL RP implants (Klockner Implant System SA, Barcelona, Spain) with a diameter of 4.1 mm and a total length of 10 mm, which were juxta-placed and equidistant in the intermentoneal region. In model 1 the implants were placed parallel to each other, whereas in model 2 the implants were placed such that there was a divergence angle of 15° between the more distal implants, and a convergence angle of 15° between the two central implants.

### 2.2. Impression Procedures

Impression procedures were performed in a room with a constant temperature range of 23–25 °C and a relative humidity of 70–80%. All impressions were performed by a single operator. To enable the better standardization of impressions, the master model remained stabilized and immobilized during the procedures (Articulated Bank, Adeo Services, Lezennes, France). A polyether adhesive (Impregum Adhesive, 3M ESPE, Seefeld, Germany) was applied to all trays and left to dry for 15 min (Figure 2). A total of 10 impressions were taken for each group, as summarized in Table 1.

**Figure 1:**
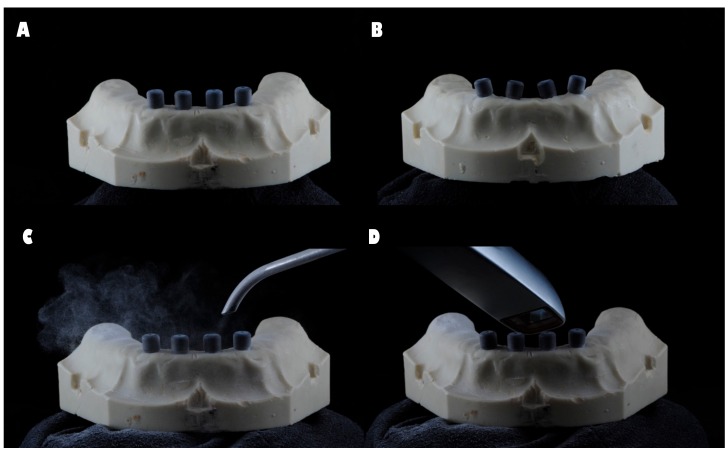
(**A**) Master model parallel scanbodies; (**B**) master model non-parallel scanbodies; (**C**) 3M™ True Definition Scanner system scanning process—powdering the model with a light dusting of titanium dioxide; (**D**) 3M™ True Definition Scanner system scanning process—reading.

The four impression copings were splinted with Dental Floss and Pattern Resin™ LS (GC, Alsip, IL, USA) 24 h prior to the impression procedure. The resin between each implant was cut before the impression was taken and reattached with Pattern ResinTM LS, with a forge time of 15 min. This procedure was performed to compensate for the shrinkage of the material and to avoid tension between the printing copings. For groups D and G, the scanbodies were placed on the implants and registered in accordance with the manufacturer’s instructions, and the model was powdered with a light dusting of titanium dioxide (3M™ True Definition Scanner, Seefeld, Germany) (Figure 1).

For the conventional impression groups, previously prepared trays were tested and the impression material (Impregum Duosoft, 3M ESPE, Seefeld, Germany) was prepared. The impression material was then applied directly around each cap in the reference model, while filling the individual tray in accordance with the manufacturer’s instructions. The tray was positioned on the model, which was kept stable by the built-in internal caps and left until complete polymerization of the material had occurred, which was designed to be approximately 6 min.

For groups A and E the impression was removed, after which the impression coping was unscrewed from each implant then screwed to an implant analog. The analog-coping assembly was fitted into the impression in the corresponding position. For groups B, C, and F, after unscrewing the impression coping from each implant and removing the tray from the reference model, an implant analog was screwed to the coping in order to stabilize the assembly between the analog and the coping.

**Figure 2:**
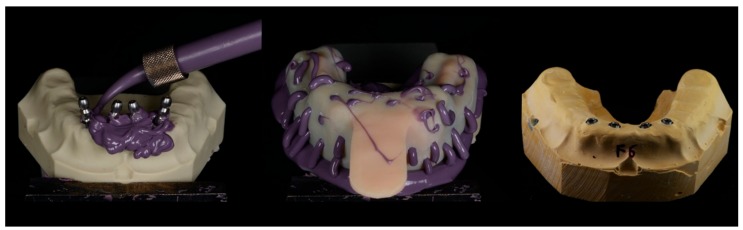
Impression protocol—obtaining study models.

**Figure 3:**
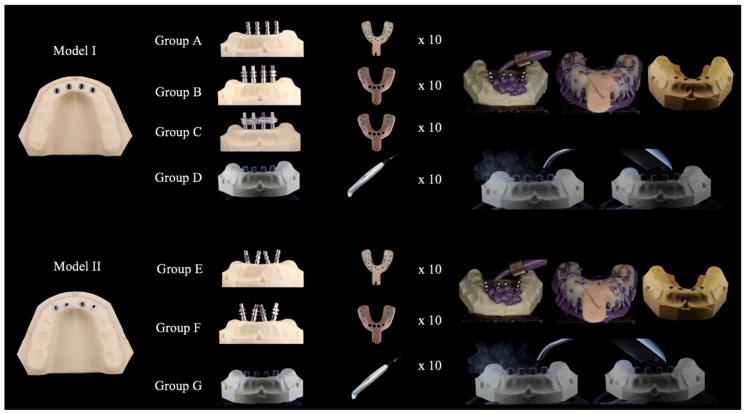
Impression protocol—obtaining study models.

All impressions were incubated at room temperature for 24 h. In order to obtain the study models, type IV plaster (Fuji-Rock, GC Europe N.V., Leuven, Germany) was prepared in accordance with the manufacturer’s instructions. The mixture was prepared in a vacuum machine (Whip-mix, Whip Mix Corporation, Louisville, KY, USA) for 1 min, which was then casted over each impression taken. In order to guarantee the complete setting of the plaster and facilitate the separation of the impression from the mold, the samples were left for 1 h (Figure 2 and Figure 3).

### 2.3. Measurement

**Figure 4:**
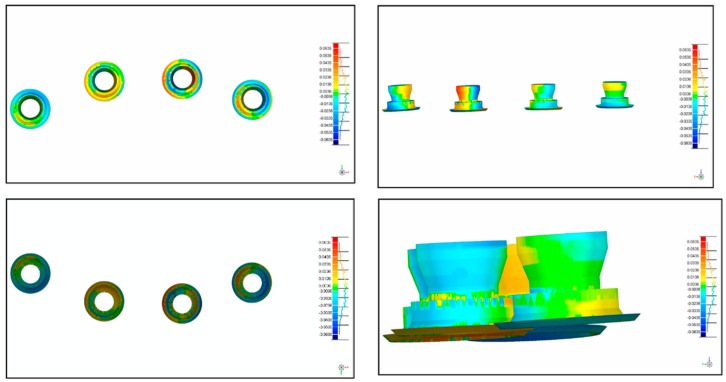
STL files overlapped using the Geomagic software (Geomagic Qualify 12, 3D Systems, Rock Hill, SC, USA), facilitating the calculation of three-dimensional (3D) deviations between the master model data and the different groups.

**Figure 5:**
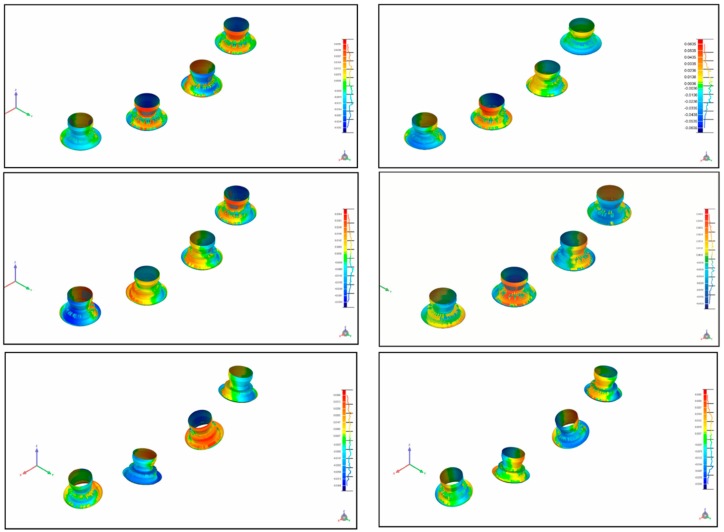

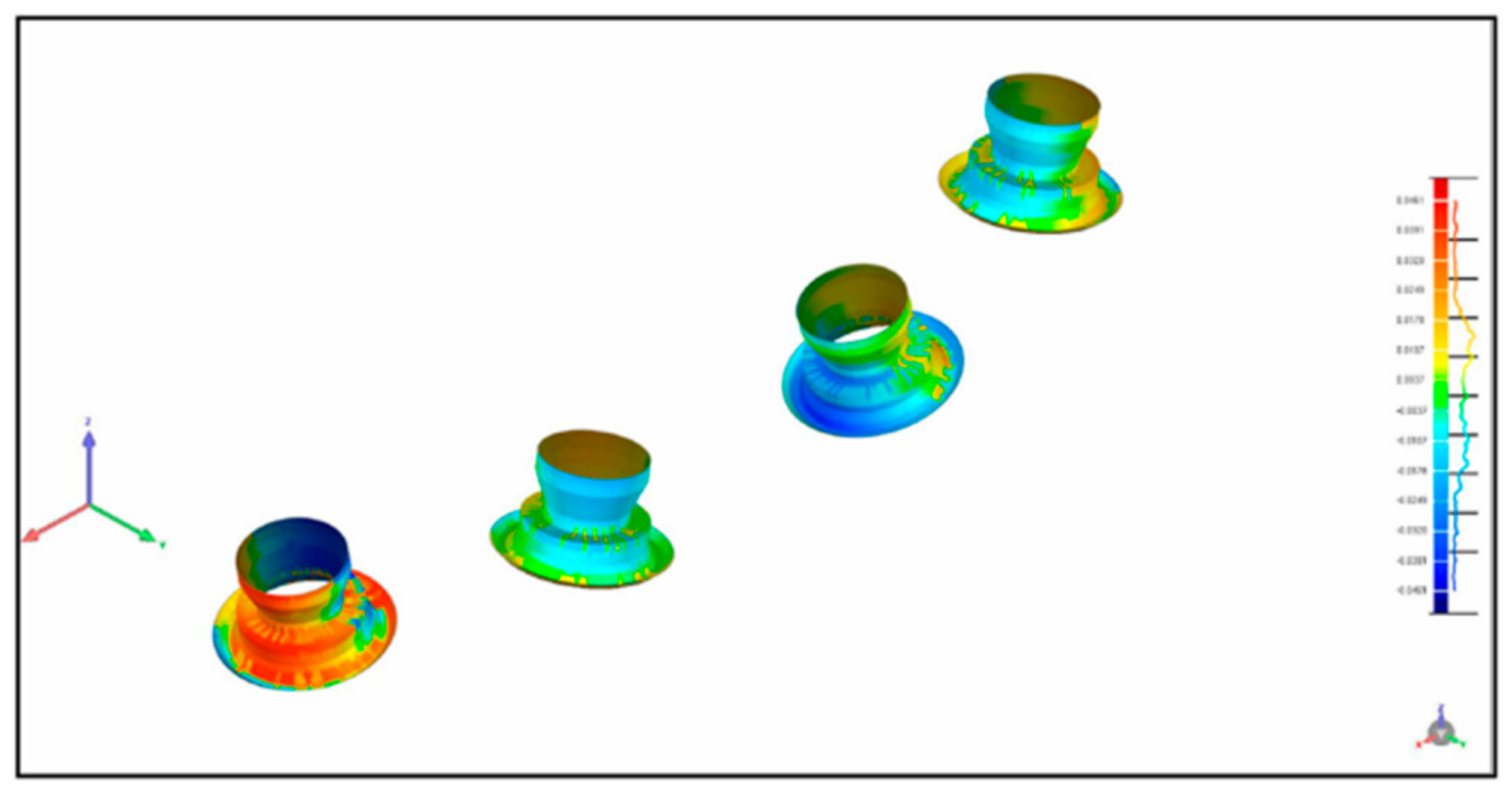
STL files of the different groups overlapped using the Geomagic software (Geomagic Qualify 12, 3D Systems, Rock Hill, SC, USA), facilitating the calculation of 3D deviations between the master model data and the different groups.

After the acquisition of the 10 digital impressions, the files were exported as SLT files for comparison. The master model and models obtained from conventional impressions were digitalized for comparison via a high-resolution extraoral scanner (Imetric IScan D104i, Porretruy, Switzerland). This scanner has very accurate tolerances of <5 μm, resulting in a near exact representation of the implant positions. For each model, the scanbodies were placed and scanned in accordance with the manufacturer’s instructions. The same procedure was repeated for each of the models used in groups A, B, C, E, and F, and for the master models. A blinded operator performed the scans for all models. The different STL files were overlapped using Geomagic software (Geomagic Qualify 12, 3D Systems, Rock Hill, SC, USA), allowing for the calculation of 3D deviations between the master model data and the different groups. The software selected 4000 aleatory coordinates in the model, 1000 in each implant (Figure 4 and Figure 5).

### 2.4. Statistics

Statistical analyses were performed using IBM Corp. Released 2016. IBM SPSS Statistics for Windows, Version 24.0. Armonk, NY: IBM Corp. The 4000 deviations in the *X*, *Y*, and *Z* axes data produced by the four techniques in the impressions in model 1 as well as by the three techniques in the impressions in model 2 were not normally distributed, as determined via the Kolmogorov-Smirnov test. Measurement of the accuracy of the various techniques and/or models could not be performed via the sum of deviations, because negative deviations cancel out positive ones, and the sum of all deviations (on a given axis) with a value of zero may not represent the best precision with regard to that axis. For this reason, we analyzed the squares of the deviations in the three axes and evaluated the median and the sum of the deviation square. It was considered that, from a mathematical perspective, the precision of an impression/model is greater when the sum of the squares of deviations is the smallest in the three axes, or, in the absence of simultaneity, the smallest in the sum of the three axes.

The square values of the deviations for the *X*, *Y*, and *Z* axes were analyzed with reference to the median and respective 25th and 75th percentiles, as well as the minimum and maximum, and the total sum. Median comparisons between three and four groups were performed via the Kruskal-Wallis test, followed by multiple comparisons adjusted using the Bonferroni method. Comparisons of medians derived from the models (two groups) were performed via the Mann-Whitney test. In all tests, *p* < 0.05 was deemed to indicate statistical significance.

## 3. Results

Two different models were prepared in order to compare the accuracy of parallel implants (model 1) and non-parallel implants (model 2). For model 1, the median values for the square deviation are shown in Table 2. The impressions in groups B and C yielded the highest median values in the *X* and *Y* axes, 36 and 33 respectively, and these median values did not differ significantly. Group C yielded significantly lower values, and group D yielded the lowest value, which differed significantly from those of the other groups. The *Z* axis exhibited a different pattern, with groups A and B exhibiting the highest values, which differed significantly, followed by group D which also exhibited values that differed significantly from the values derived from groups A and B. Group C yielded the lowest values, which differed significantly from those of the other groups. Group D, which incorporated the use of the 3M™ True Definition Scanner system, yielded significantly lower median values for the three axes as well as a smaller value of the sum of the total of the square of three deviations, indicating significantly higher precision for this group in model 1. The value for the sum in group D was 1,068,292, which was significantly lower than those of groups A (2,114,342), B (2,165,491), and C (1,265,918).

For model 2, evaluation of the precision of the impressions based on the median of the square of the deviation is shown in Table 3. Group F, with open tray impressions, yielded medians of the square of the deviation of X = 17, Y = 15, and Z = 15, deviations that were significantly lower than those of the other groups (followed by groups E then G), and the median of the total of the squares of the three deviations was also significantly lower. Group F yielded the lowest sum of the total of the square of the three deviations, indicating significantly higher precision for this group in model 2; 1,257,835. Notably, in model 2 the values did not differ as much as they did in model 1, wherein the respective values were 1,660,975 and 1,489,328 in groups E and G.

We then analyzed the differences between the two models with regard to defined types of impression techniques. Comparing groups A and E (Table 4; closed tray impressions with repositioning copings), group E yielded a smaller overall median value of the square of the deviations, suggesting higher accuracy. In a similar comparison of groups B and F (Table 5), corresponding to open tray impressions for open tray impression copings, group F also yielded a smaller overall median value of the square of the deviations, suggesting higher accuracy. Lastly, groups D and G were compared (Table 6) via the 3M™ True Definition Scanner system, and group D—incorporating a parallel conformation—yielded a smaller overall median value of the square of the deviations, suggesting higher accuracy.

## 4. Discussion

The in vitro data generated in the current study suggest that it is technically possible to fulfill this requirement via existing technology. Notably, however, numerous factors pertaining to the oral cavity can influence the accuracy of optical impressions, including lack of space, patient movement, and saliva flow [27]. Therefore, in vivo studies investigating full-arch impressions acquired via digital intraoral impression techniques are necessary.

Some errors may be introduced during any of the several clinical procedures required, such as improper connection of the relevant components, excessive dimensional changes of the impression materials, minor movements caused by unscrewing of the impression copings, and subsequent screwing of the implant analogs. The clinical significance of the associated distortion magnitude remains controversial [28].

Drawing comparisons between the results of the present study and those of previous studies is problematic, because different digitization and evaluation methods were used. Brosky et al. [29] used a method in which the differences between two models were graphically displayed and the area under this deviation curve was calculated. They reported deviations were between 27 and 297 μm among models of conventional impressions with polyvinyl siloxane and the reference model. However, the study lacked an evaluation of the measuring precision of the scanner used.

In general, it has been established that a well-fitting prosthesis is important to avoid complications and assure the longevity of the construction. However, this is not easy to achieve. The application of CAD/CAM in dentistry has improved the accuracy of frameworks compared to conventionally cast frames [30]. Despite this improvement, implant-supported frameworks have still exhibited microgaps as large as 38 μm, depending on the span of the construction [31]. The remaining misfit is largely caused by errors that occur during the impression process and the production of the stone cast [32,33]. In an effort to eliminate these errors, the concept of digital impressions has been introduced. 

When evaluating the fit of a tooth-supported crown, digital impressions resulted in a better fit than conventional impressions and stone casts for single crowns [34,35], as well as for fixed partial dentures [35]. Although the use of digital impressions can clearly improve the framework fit of tooth-supported restorations, its final accuracy still varies, depending on the shape of the preparations and the span of the framework. Little research has been conducted on the use of intraoral scanners for the full arch. Using a full-arch model containing 14 tooth preparations, Patzelt et al. [36] tested the precision of four intraoral scanners. The trueness values were between 38.0 and 332.9 μm, while the precision ranged from 37.9 to 99.1 μm. The authors concluded that only one intraoral scanner (Lava C.O.S.) could be recommended for use in edentulous jaws [36].

Ender and Mehl [37] evaluated several conventional impression materials and intraoral scanners. The digital impressions of a full arch yielded values between 29 and 45 μm, and accuracy ranged from 19 to 63 μm, which was not significantly better than the conventional impressions. The authors stated that the intraoral scanners demonstrated more local deviations and that their accuracy depended largely on the scanning technique. Su and Sun [37] evaluated the precision of the Trios scanner (TRIOS, 3shape, København, Denmark) and compared it with a laboratory scanner. Not only was the precision of the intraoral scanner significantly lower, but also the deviation increased with the number of teeth scanned.

Mangano FG et al. [21] compared the trueness and precision of four intraoral scanners (Trios^®^ (3-Shape, Copenhagen, Denmark); CS 3600^®^ (Carestream, Rochester, NY, USA); Zfx Intrascan^®^ (Zfx Birmingham, UK); Planscan^®^ (Planmeca Romexis, Helsinki, Finland)). In this in vitro study they compared the trueness and precision of four intraoral scanners in oral implantology, using two models (a partially edentulous patient with three implants, and a totally edentulous patient with six implants). Although no differences in trueness and precision were found between the partially and totally edentulous models, the investigated digital impression systems differed significantly [21].

These results are not concordant with the findings of Imburgia et al. [20] In their in vitro study, they compared the accuracy of four of the latest generation intraoral scanners (CS3600^®^ (Carestream, Rochester, NY, USA), Trios3^®^ (3-Shape, Copenhagen, Denmark), Omnicam^®^ (Sirona, Bensheim, Germany), TrueDefinition^®^ (3M Espe, St. Paul, MN, USA)) in two different situations (in a partially edentulous model with three implants and in a fully edentulous model with six implants, respectively). Excellent results in terms of accuracy were achieved with all intraoral scanners, scanning the two different models. However, the scanning accuracy was higher in the partially edentulous model than in the fully edentulous model. This indicates that, despite the considerable progress made by the latest generation scanners, scanning a fully edentulous patient remains more difficult than scanning an area of more limited extent, and consequently the design and milling of full-arch restorations on the basis of these scanning data may still present problems [22].

Although the statistical method applied in the current study differed from those of most previous studies utilizing similar experimental methodology, the statistical tendencies of the results pertaining to digital impression groups vs. conventional printing groups (see Table 2 and Table 3) were concordant with those of numerous previous studies [13,36,37,38]. 

Angulation did not seem to be a determining factor in the precision of the models investigated, because the impressions derived from model 2 yielded slightly better results than those derived from model 1 (see Table 2 and Table 3). This is concordant with the findings of Gimenez et al. [38], who reported that with angulations of up to 30°, implant angulation did not affect accuracy statistically significantly when a blue light LAVA C.O.S. scanner (active wavefront sampling technology) (TRIOS, 3shape, København, Denmark) was used. Additionally, in a similar study by the same group utilizing an identical angulated implant design, it was reported that an implant angulation of up to 30° did not statistically significantly affect the accuracy of digital impressions when a red light iTero scanner (Align Technology Inc., San Jose, CA, USA) with parallel confocal imaging was used [39]. The common denominator in both studies was that operator experience may play a role in the accuracy of digital impressions and that a learning curve exists until the clinician becomes sufficiently proficient with regard to the operation of digital impression scanners [39,40].

Acquiring digital implant impressions may currently be even more challenging than acquiring impressions via a laboratory scanner. Scanning of a single implant can be achieved with high predictability, as has been shown in several previous studies and case reports [41,42,43,44]. When using a monolithic restoration, a complete digital workflow is possible because the abutment and crown are virtually designed and manufactured in their final shape [44]. However, when scanning multiple implants in an edentulous jaw, some difficulties may arise. As multiple, identical scanbodies are used, it may be difficult for the intraoral scanner to distinguish one from another and thus identify the correct location in the jaw. Intraoral scanners that work with a photosystem may paste images of different scanbodies on top of each other [45].

Papaspyridakos et al. [15] compared a digital impression system (Trios) with several conventional (polyether splinted and non-splinted) impression techniques for the registration of five implants in edentulous mandibles. They reported that there were no significant differences between the digital and conventional impression methods, and concluded that digital impressions could be used for implant impressions in edentulous jaws. This could be explained by differences in the number of implants, a different implant connection, or a different design and fit of the scanbody. Conventional extra-oral scanbodies were used in that previous study, which were significantly longer than those used in the current study. As reported by Fluegge et al. [45], precision decreases when shorter and smaller (intraoral) scanbodies are used. Stimmelmayr et al. [46] reported a significant difference in scanbody fit between the actual original implants and laboratory analogs, in favor of the latter.

While the results of the current in vitro study are very promising, they did entail some limitations regarding oral cavity scanning. Scanning in the mouth may involve double the error compared to scanning a model, due to the different environment [47]. Another difference between in vivo and in vitro scanning is the stability of the scanning surface. The shape of the mucosa may change depending on jaw movements, which complicates the scanning procedure because it depends on the presence of fixed reference points [45]. Similarly, a larger inter-implant distance combined with a flat mucosal surface may result in a lack of reference points to enable correct stitching [40]. In the current study, the implants were positioned relatively close to each other. It can be assumed that if the inter-implant distance is increased, the scanning process would become more difficult, which may reduce accuracy.

There are different intraoral scanning systems. While some use powder others do not, and according to the existing literature [13,36], the need for powder and opacification is typical of the first-generation intra oral scanners; the more recently introduced devices can detect optical impressions without using powder [21,48,49]. Technically, a scanner that allows the clinician to work without opacification should be preferred, as powder may represent an inconvenience for the patient [21,48,49]. In addition, applying a uniform layer of powder is complex [21,48,49]. An inappropriate opacification technique may result in layers of different thicknesses at various points of the teeth, with the risk of errors that reduce the overall quality of the scan [21,48,49].

Digital implant dentistry is increasing in popularity and exhibits good potential; however, further studies are needed to assess and compare the clinical accuracy of digital vs. conventional implant impression techniques in both partially and completely edentulous patients. Additionally, the complete digital workflow from planning to definitive rehabilitation should be assessed and compared with conventional methods in terms of time efficiency, learning curves, accuracy, and economic aspects. In clinical practice, the combined utilization of both the digital and the conventional approach may yield additional advantages specific to each individual case.

## 5. Conclusions

Under the limitations of the present in vitro study, the following conclusions may be drawn:

For a model with four parallel implants, the deviations of the digital impressions were smaller than those associated with the conventional techniques. This improvement was not observed when using a model with four angled implants, however, where the conventional techniques yielded similar results. Therefore, digital impressions of full-arch models were able to achieve the accuracy of conventional impressions in an in vitro model.

However, further in vivo studies are needed to confirm the in vitro results.

## Figures and Tables

**Table 1:** Descriptions of the different impression techniques and models used, with their corresponding nomenclatures.

Impression System	Model 1	Model 2
Closed tray impressions with replacement abutments	A	E
Open tray impression groups for dragging copings, without splinting	B	F
Open tray impressions for ferrules	C	-
Using the 3M™ True Definition Scanner	D	G

**Table 2:** Model 1 (without angulation). Comparison of the values of the square of the X-deviation, Y-deviation, Z-deviation, and the total in the impressions of groups A (closed tray impressions with replacement abutments), B (open tray impressions for dragging copings, without splitting), C (open tray impressions for ferrules), and D (using the 3M™ True Definition Scanner).

Axis	Group	Median (P25–P75)		*p*	Min–Max	Sum
***X***	A	20 (1–138)	b	<0.001	0–6579	758,315
	B	36 (2–235)	a		0–5165	914,706
	C	33 (1–204)	a		0–3737	693,878
	D	15 (1–121)	c		0–1791	507,725
***Y***	A	22 (2–113)	c	<0.001	0–4456	520,289
	B	36 (2–197)	a		0–5387	714,484
	C	26 (2–104)	b		0–1280	332,341
	D	17 (1–97)	d		0–1177	341,292
***Z***	A	26 (1–180)	a	<0.001	0–5797	835,738
	B	21 (1–113)	b		0–8615	536,301
	C	11 (1–60)	d		0–1982	239,699
	D	15 (1–75)	c		0–808	219,275
**Total**	A	194 (51–623)	b	<0.001	0–9909	2,114,342
	B	242 (60–705)	a		0–10,943	2,165,491
	C	163 (43–446)	c		0–4226	1,265,918
	D	128 (32–383)	d		0–2276	1,068,292

a, b, c, d: different lowercase letters indicate significant differences between the medians of the groups (a = highest median).

**Table 3:** Model 2 (with angulation). Comparison of the values of the square of the X-deviation, Y-deviation, Z-deviation, and the total in the impressions of groups E (closed tray impressions with replacement abutments), F (open tray impressions for drag copings without splinting), and G (using the 3M™ True Definition Scanner).

Axis	Group	Median (P25–P75)		*p*	Min–Max	Sum
***X***	E	26 (4–128)	b	<0.001	0–4376	547,692
	F	17 (2–107)	c		0–4730	601,235
	G	32 (5–170)	a		0–3277	781,324
***Y***	E	21 (1–121)	b	<0.001	0–3634	444,326
	F	15 (1–86)	c		0–3794	384,520
	G	22 (2–90)	a		0–1441	298,488
***Z***	E	24 (3–136)	b	<0.001	0–5755	668,958
	F	15 (2–73)	c		0–1969	272,080
	G	32 (3–132)	a		0–1402	409,517
**Total**	E	191 (51–521)	a	<0.001	0–7016	1,660,975
	F	129 (32–325)	c		0–5920	1,257,835
	G	177 (53–440)	b		0–3654	1,489,328

a, b, c: different lowercase letters indicate significant differences between the medians of the groups (a = highest median).

**Table 4:** Comparison of the values of the square of the X-deviation, Y-deviation, Z-deviation, and the total in the impressions of the groups of closed tray impression with replacement abutments, without and with angulation (groups A and E, respectively).

Axis		Median (P25–P75)		*p*	Sum
***X***	model 1-A	20 (1–138)	b	<0.001	758,315
	model 2-E	26 (4–128)	a		547,692
***Y***	model 1-A	22 (2–113)	a	<0.001	520,289
	model 2-E	21 (1–121)	b		444,326
***Z***	model 1-A	26 (1–180)	a	<0.001	835,738
	model 2-E	24 (3–136)	b		668,958
**Total**	model 1-A	194 (51–623)	a	<0.001	2,114,342
	model 2-E	191 (51–521)	b		1,660,975

a, b: different lowercase letters indicate significant differences between the medians of the groups (a = highest median).

**Table 5:** Comparison of the values of the square of the X-deviation, Y-deviation, Z-deviation, and the total in the impressions of the open tray impression groups for dragging copings, without splitting, and with and without angulation (groups B and F, respectively).

Axis		Median (P25–P75)		*p*	Sum
***X***	model 1-B	36 (2–235)	a	<0.001	914,706
	model 2-F	17 (2–107)	b		601,235
***Y***	model 1-B	36 (2–197)	a	<0.001	714,484
	model 2-F	15 (1–86)	b		384,520
***Z***	model 1-B	21 (1–113)	a	<0.001	536,301
	model 2-F	15 (2–73)	b		272,080
**Total**	model 1-B	242 (60–705)	a	<0.001	2,165,491
	model 2-F	129 (32–325)	b		1,257,835

a, b: different lowercase letters indicate significant differences between the medians of the groups (a = highest median).

**Table 6:** Comparison of the values of the square of the X-deviation, Y-deviation, Z-deviation, and the total in the impressions of the groups using the 3M™ True Definition Scanner, without and with angulation (groups D and G, respectively).

Axis		Median (P25–P75)		*p*	Sum
***X***	model 1-D	15 (1–121)	b	<0.001	507,725
	model 2-G	32 (5–170)	a		781,324
***Y***	model 1-D	17 (1–97)	b	<0.001	341,292
	model 2-G	22 (2–90)	a		298,488
***Z***	model 1-D	15 (1–75)	b	<0.001	219,275
	model 2-G	32 (3–132)	a		409,517
**Total**	model 1-D	128 (32–383)	b	<0.001	1,068,292
	model 2-G	177 (53–440)	a		1,489,328

a, b: different lowercase letters indicate significant differences between the medians of the groups (a = highest median).
